# Useful diagnostic histogenetic features of ectopic odontogenic ghost cell tumours

**DOI:** 10.1186/s12903-022-02169-3

**Published:** 2022-04-20

**Authors:** Yuri Noda, Chisato Ohe, Mitsuaki Ishida, Kimiaki Okano, Kaori Sando, Naoya Hada, Yusuke Ebisu, Takuo Fujisawa, Masao Yagi, Hiroshi Iwai, Koji Tsuta

**Affiliations:** 1 Department of Pathology and Laboratory Medicine, Kansai Medical University Hirakata Hospital, 2-3-1 Shin-machi, Hirakata, Osaka 573-1191 Japan; 2 Department of Otolaryngology, Head and Neck Surgery, Kansai Medical University Hirakata Hospital, 2-3-1 Shin-machi, Hirakata, Osaka 573-1191 Japan

**Keywords:** Dentinogenic ghost cell tumour, Ghost cell, Odontogenic tumour, Calcifying odontogenic cyst, *CTNNB1* mutation

## Abstract

**Background:**

Ectopic odontogenic tumours are rare and difficult to diagnose. Consequently, they are occasionally misdiagnosed as other tumours and overtreated. Dentinogenic ghost cell tumours (DGCTs) are odontogenic neoplasms characterised by a *CTNNB1* mutation, ghost cell appearance, and dentinoid-like calcification. Herein, we present a case of ectopic DGCT on the floor of a patient’s mouth, providing reliable clinicopathological and genetic evidence of its odontogenicity for the first time.

**Case presentation:**

A 72-year-old man presented with painless sublingual swelling. Imaging revealed a multi-lobulated, solid-cystic mass on the floor of his mouth. Cytological evaluation showed folded epithelial clusters composed of basaloid cells, keratinised material, and calcification. Histological analysis revealed a multi-cystic, cribriform to solid nest, with an odontogenic satellate reticulum-like epithelium, including ghost cells and dentinoid matrix deposition. Immunohistochemical analysis found that CK19, CK5/6, bcl-2, and p63 were diffuse positive, β-catenin was focal positive in the nuclei, and the cells in the dentinoid matrix were positive for DMP1. The *CTNTTB1* mutation was detected, leading to the final diagnosis of ectopic DGCT. There was no recurrence during the 6-month follow-up.

**Conclusions:**

Overall, we have presented a comprehensive clinical overview of DGCT and identified its pathological and genetic features. This report will aid in the recognition of this rare disease in the future and help to avoid misdiagnosis and overtreatment.

## Background

Odontogenic tumours arising from extra-alveolar sites are extremely rare and occasionally misdiagnosed as other tumours and overtreated [1–4]. Three types of odontogenic ghost cell lesions can occur in the oral cavity: dentinogenic ghost cell tumour (DGCT), calcifying odontogenic cyst (COC), and ghost cell odontogenic carcinoma (GCOC) [1, 2]. Only one extra-alveolar (ectopic) odontogenic ghost cell tumour case has been reported to date [3]; thus, owing to its rarity, little is known about this disease. Moreover, in the absence of any conclusive oncogenic, histopathologic, and genetic evidence, it is unclear whether ectopic odontogenic ghost cell tumours have an odontogenic origin; furthermore, information on tumour behavior is also limited.

DGCTs are classified as a group of lesions with ghost cells with a *CTNNB1* mutation, along with COC and GCOC. However, tumours with *CTNNB1* mutations have similar histological structures and range from benign to malignant. Although the differential diagnosis of DGCT is important, it is difficult, especially if the tumour is of ectopic origin.

We present the case of a patient with an ectopic DGCT on the floor of their mouth. To the best of our knowledge, this is the first report of ectopic DGCT accompanied by reliable clinicopathological evidence of its odontogenic origin. We also identify its radiological features such as solid-cystic mass and characteristic histopathological odontogenic features such as the presence of dentinoid-like calcifications and stellate epithelial islands containing ghost cells with a *CTNNB1* mutation [1–12]. We believe that this report could help to facilitate the accurate diagnosis of this rare disease in the future.

## Case presentation

**Fig. 1:**
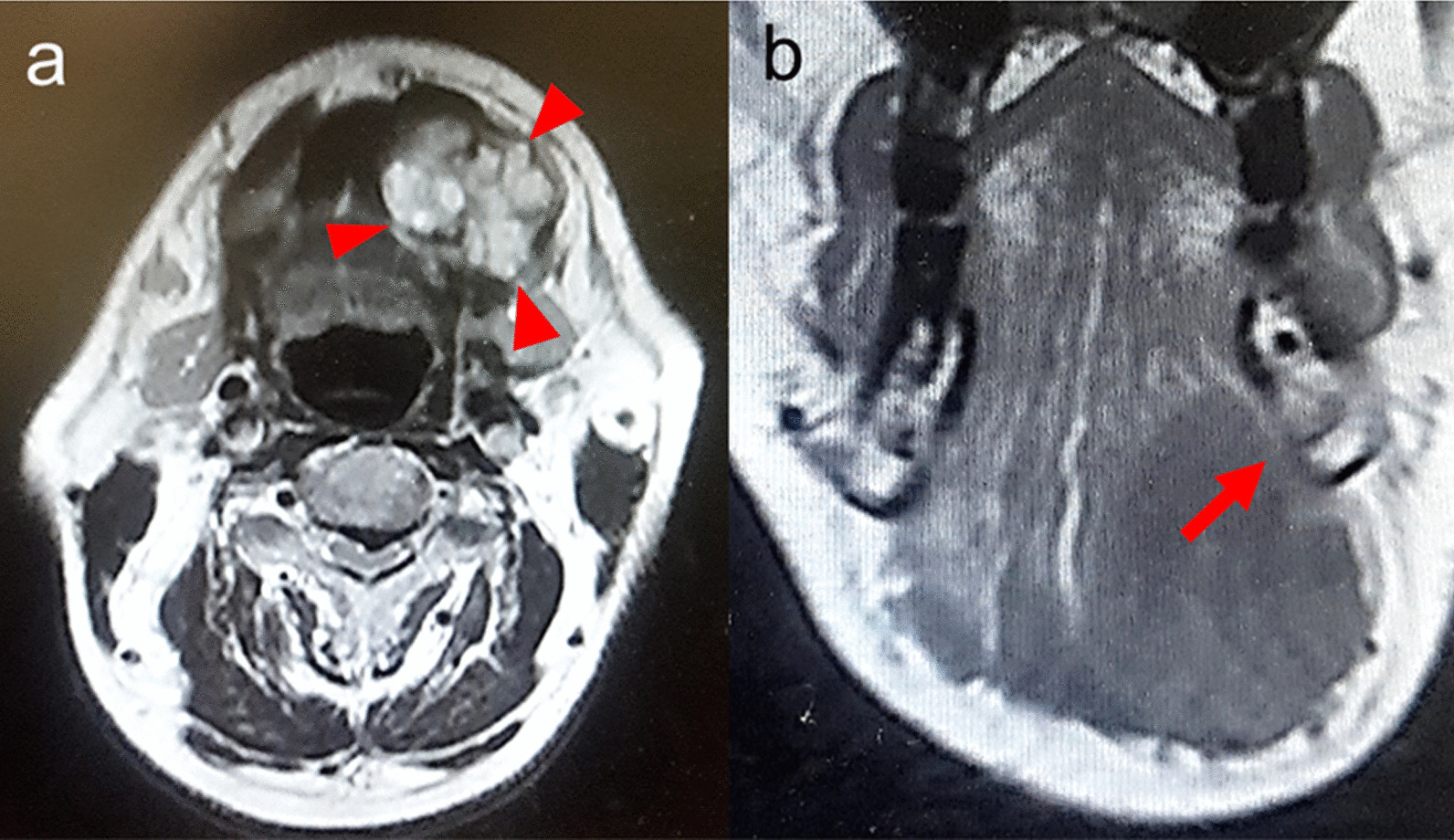
Magnetic resonance imaging. **a** Magnetic resonance imaging (T1-weighted image) revealed a well-circumscribed lobulated mass on the left side of the floor of the oral mouth (arrowhead). **b** A frontal section (T2-weighted image) shows an expanded mass adjacent to the mandible without connection to the gingival mucosa or jawbone (arrow)

A 72-year-old Japanese man with no remarkable medical or family history presented with painless sublingual swelling identified during a follow-up examination for a myocardial infarction. Clinical examination revealed an elastic mass in the sublingual area covered by normal mucosa. Magnetic resonance imaging (MRI) showed a well-circumscribed lobulated, multi-cystic solid mass located on the floor of the mouth (Fig. 1a). There was no connection between the mass and the gingiva or jawbone (Fig. 1b).

**Fig. 2:**
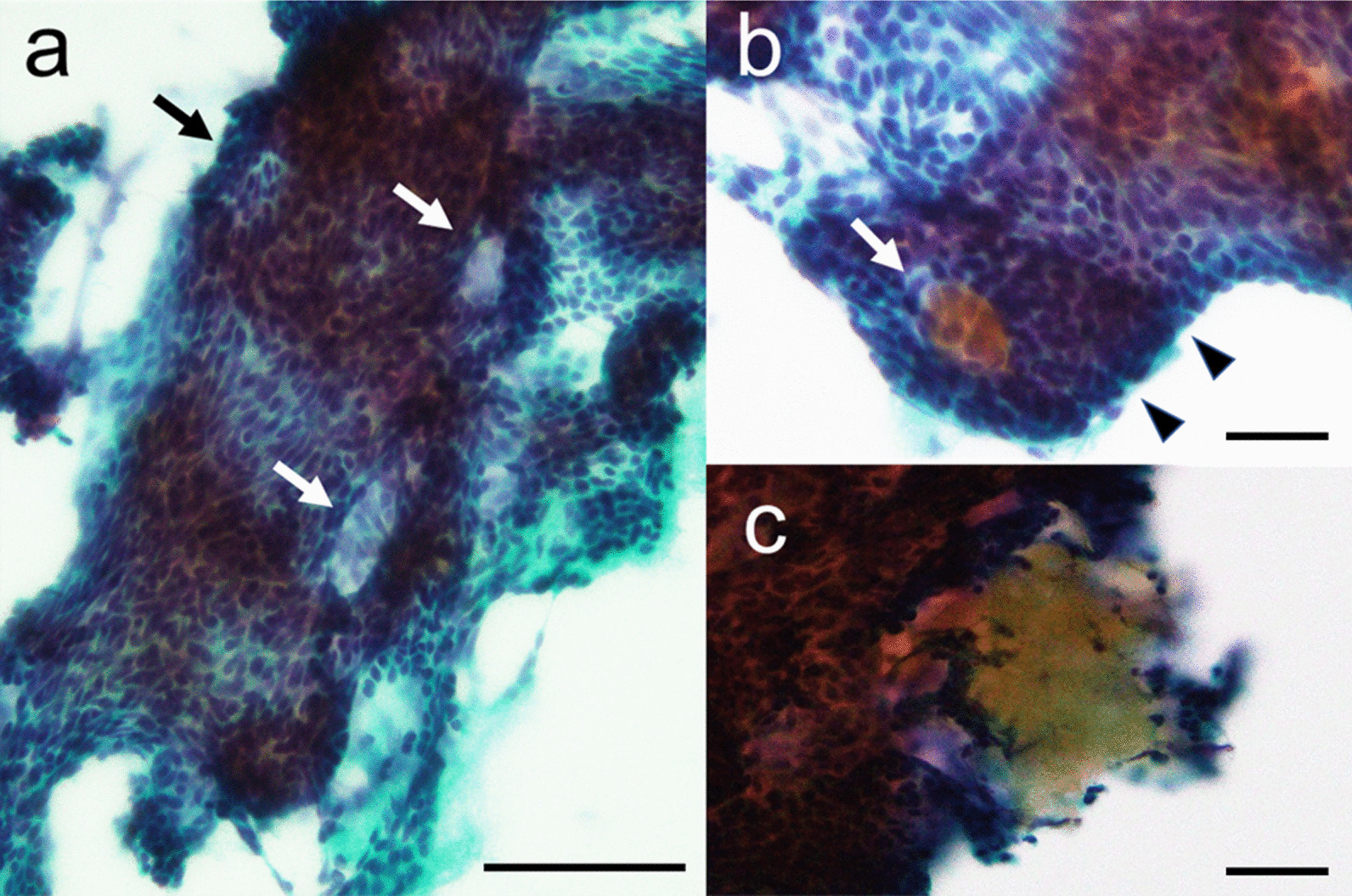
Cytological findings. **a** Fine needle aspiration revealed folded epithelial clusters including duct-like formations (arrows; Papanicolaou staining). **b** Clusters consisting of basaloid cells with admixed orange G-positive, round structures lacking nuclei (arrows), and peripheral palisading (arrowhead; Papanicolaou stain). **c** Calcified material (Papanicolaou staining). Scale bar = 100 µm (**a**), 50 µm (**b**, **c**). Images were taken using an OLYMPUA BX53 and scaled with PLYMPUS cellSens Standard

Fine needle aspiration showed folded epithelial clusters with duct-like formations (Fig. 2a). These clusters consisted of basaloid cells lacking prominent nuclear atypia and admixed orange G-positive, round structures lacking nuclei. Peripheral palisading and some calcified materials were observed (Fig. 2b, c). Cytologically, a basaloid tumour such as basal cell adenoma or adenocarcinoma was suspected, and a diagnosis of “atypia of undetermined significance” was considered. Based on the location and cytological features, a sublingual tumour was suspected, and tumour excision and level I lymph node dissection were performed. The intraoperative analysis revealed a circumscribed mass with no connection to the alveolar bone or oral floor mucosa.

The resection margin was tumour-free, and no additional treatment was performed postoperatively. The patient was followed up for 6 months, with no sign of recurrence when assessed using MRI.

**Fig. 3:**
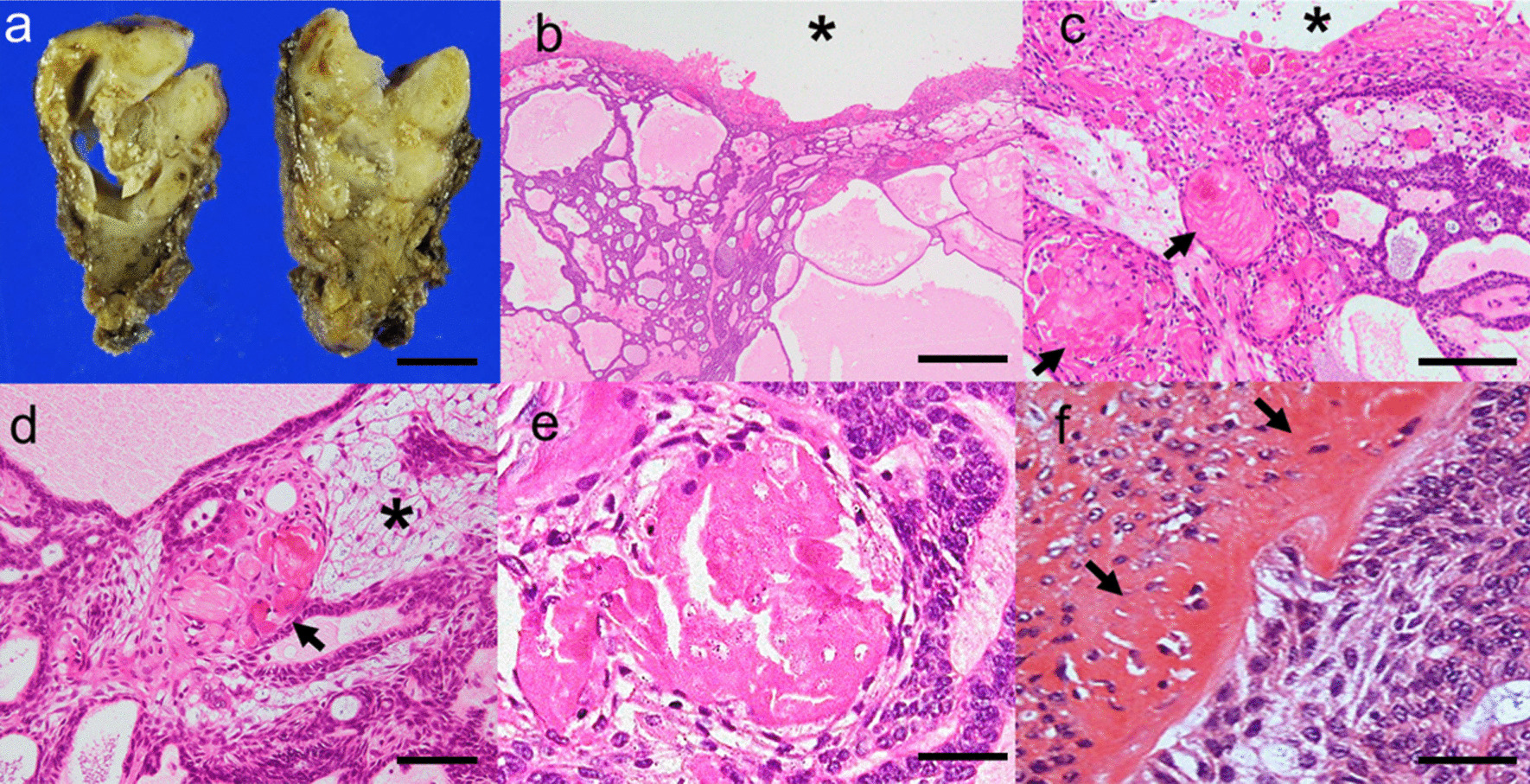
Macroscopical and histological findings. **a** The surgical specimen was a tan-white, lobulated solid cystic mass. **b**, **c** The cystic space was lined with a thick wall containing polygonal epithelial cells and ghost cells (arrows), similar to a calcifying odontogenic cyst, and translated the cribriform or plexiform basaloid component (asterisk, cystic space; hematoxylin and eosin [HE] staining). **d** The tumour nest with satellate reticulum-like cells including ghost cells and peripheral palisading (asterisk, satellate reticulum like nest; arrows, ghost cells; HE staining). **e** Ghost cells with prominent eosinophilic homogenisation and an absence of nuclei (HE staining). **f** Dentinoid material deposition adjacent to the tumour nest (arrows, dentinoid material; HE staining). Scale bar = 1 cm (**a**), 500 µm (**b**), 200 µm (**c**), 100 µm (**d**), and 50 µm (**e**, **f**). Images were taken using an OLYMPUA BX53 and scaled with PLYMPUS cellSens Standard

**Fig. 4:**
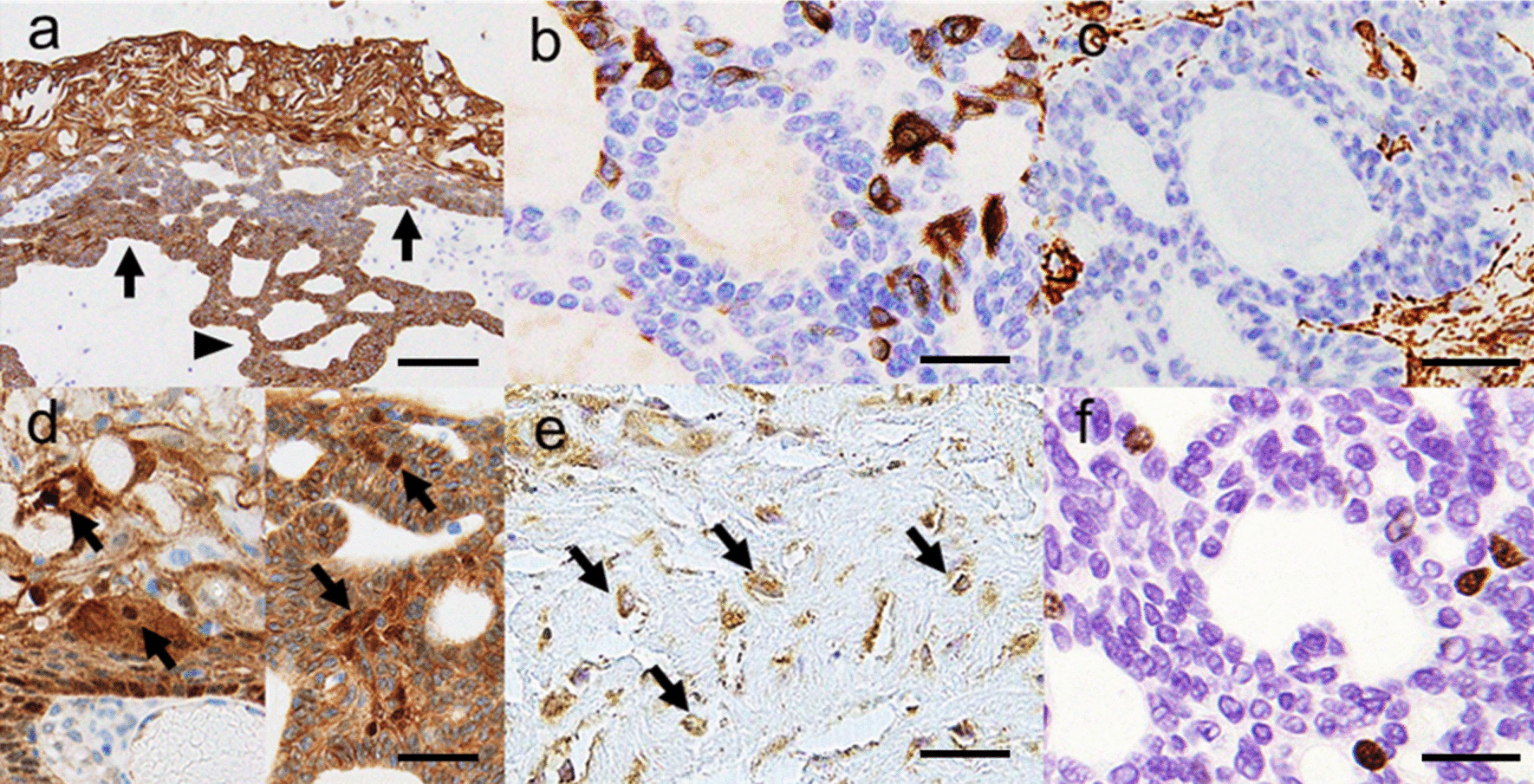
Immunohistochemical features. **a** CK19 was diffusely positive in both the cyst wall and solid nest (arrows, cysts wall; arrowheads, solid nest). **b** CK7 was focal positive indicating that there were no duct structures. **c** SMA was negative in the solid nest and stromal cells, whereas the internal control was positive. **d** Nuclear accumulation of β-catenin was detected (arrows, positive cells; left figure, cyst wall component; right figure, solid nest). **e** Dentin matrix protein-1 was positive in the cell nuclei with dentinoid material deposition (arrows, positive cells). **f** The Ki-67 index was low. Scale bar = 500 µm (**a**), 50 µm (**b**–**f**). Images were taken using an OLYMPUA BX53 and scaled with PLYMPUS cellSens Standard

The surgical specimen was a tan-white, elastic, lobulated solid mass, with multiple small cystic spaces (Fig. 3a). Histological examination revealed a multi-cystic, solid mass surrounded by a thin fibrous capsule. The cyst lining of variable thickness was composed of squamous-to-polygonal epithelial cells, which translated to the plexiform and cribriform components (Fig. 3b). Both the cyst and solid components had prominent large, homogenised eosinophilic cells with or without nuclei (Fig. 3c–e). The tumour nest showed basaloid cell proliferation with peripheral palisading and a pale central epithelium, similar to that of the stellate reticulum adjacent to the dentinoid material deposition (Fig. 3d, e). Tumour cells showed hyperchromatic nuclei with mild atypia and mitoses (2/10HPF). There was no invasion of the adjacent salivary gland, adipose tissue, lymphovascular, or perineural structures. In the gland-like structure, Alcian blue staining showed focal positivity, whereas d-PAS was negative. Immunohistochemically, CK19, CK5/6, bcl-2, and p63 were diffusely positive in the cyst wall and solid nest (Fig. 4a). Immunostaining for ductal markers, such as CK7, was focal positive in the solid nest and cyst wall, whereas that for CEA was negative (Fig. 4b). Myoepithelial cell markers such as S-100, SMA, GCDFP, and WT1 were absent (Fig. 4c). Nuclear accumulation of β-catenin was detected in the cyst wall and solid nest (Fig. 4d), and cell nuclei comprising dentinoid material deposition were positive for dentin matrix protein-1 (DMP1), suggesting osteoblastic differentiation in the mineralised bone or dentin (Fig. 4e) [1, 2, 13]. The Ki-67 index was 5% (Fig. 4f). These immunohistochemical results showed that there were no true ducts composed of ductal cells and myoepithelial or basaloid cells to suggest a salivary gland origin. The hyalinised matrix showed characteristic odontogenic calcification. Next-generation sequencing (AmpliSeq Cancer Hotspot Panel V2) revealed a missense point mutation in *CTNNB1* (p.Ile35Ser, c.104T>G). A final diagnosis of DGCT on the floor of the mouth was established.

## Discussion and conclusions

Ectopic odontogenic tumours are rare, and to the best of our knowledge, only one ectopic DGCT case has been described [3]; additionally, no extra-oral or ectopic DGCTs have been reported. Odontogenic ghost cell lesions, originally described by Ide et al. [14], are comprised of COC, DGCT, and GCOC; these three odontogenic ghost cell lesions form an analogous group with obligate ghost cell differentiation associated with *CTNNB1* mutations [1, 2, 8, 9]. They can be classified as central (intraosseous) or peripheral (gingival or alveolar mucosal) based on their clinical presentation [1, 2].

Clinically, most DGCTs occur in the jaw bone (maxilla:mandible = 1:1) and show benign but locally infiltrating behavior [1, 2]. They are more common in men (M:F = 2:1), and usually occur in the fifth decade of life (range 0–89 years, mean: 47.0 years) [15]. Patients usually complain of progressive or slow-growing nodules with swelling, with or without pain [1, 16]. Radiologically, DGCT shows a cystic or solid mass with calcification [17]. In the present case, although the patient was older than the mean age for DGCT occurrence and the mass was found as a sublingual gland mass without connection to the alveolar tissue, as determined by radiological and operative findings, the other clinical findings were consistent with the DGCTs that are typically reported [1, 2, 17].

To the best of our knowledge, this is the first report to meticulously describe the cytological findings in relation to DGCT. The cell cluster exhibited basaloid cell proliferation with peripheral palisading. These findings are consistent with those of basal cell adenoma/adenocarcinoma, and we also suspected salivary gland tumours. However, calcification and admixed orange G-positive structures without nuclei, similar to dentino-osteoid materials and ghost cells, are a differential feature and thus an important cytological feature of DGCTs.

The histological features of DGCTs include: odontogenic histological features such basaloid to polygonal cell proliferation, ameloblastoma-like epithelial nests resembling the stellate reticulum including aberrant keratinisation regarded as ghost cells, the deposition of immature to mature dentinoid or dentino-osteoid materials, and occasional association with a COC-like cystic component [1, 2]. The ghost cells have enlarged, polygonal eosinophilic cytoplasms without nuclei [1, 2]; however, a faint outline of the absent nucleus may be seen. These histological features indicate the odontogenic nature of the tumour and are important for diagnosis. The neoplastic cells are strongly positive for cytokeratin AE1/3, CK5, CK7, CK14, and CK19, but negative for vimentin, desmin, SMA, and CD34. The Ki-67 index has been reported to be < 5% [1, 2]. These histological findings are consistent with those of the case presented here. Moreover, the positive staining of DMP1 in the cells comprised of hyalinised material suggested osteoblastic differentiation, dentinoid matrix production as in odontogenic tumours, and no deposition of the basement-membrane-like material as in salivary gland tumours [1, 2, 13].

In addition to these histological findings, a *CTNNB1* mutation was also detected in the present case. Ghost cell differentiation [12], *CTNNB1* mutations, and nuclear β-catenin accumulation are all detected in the histologically analogous group of basaloid lesions including COC, DGCT, and GCOC [6, 7], basal cell carcinoma [18, 19], basal cell adenoma/adenocarcinoma [1, 2], pilomatrixoma, pilomatrical carcinoma [8, 9], and adamantinomatous craniopharyngioma [10]. The *CTNNB1* mutations elicit differentiation of the basaloid tumour cells into hair-like cells called ghost cells [6, 20, 21]. However, except for DGCTs, tumours with ghost cell differentiation lack characteristic odontogenic histopathological features such as stellate reticulum-like epithelium, association with the COC-like cystic lesion, and dentinoid matrix deposition. Considering the anatomical site and histogenetic features, the important differential diagnoses in the present case were basal cell adenoma/adenocarcinoma and basal cell carcinoma with ghost cell differentiation, which is a recently proposed entity [5, 11, 12]. However, basal cell adenoma/adenocarcinoma exhibits two-cell morphology consisting of CK7-positive ductal structures and p63-, SMA-, CK5/6-, WT-1-, or podoplanin-positive myoepithelial/basal cell components. Basal cell carcinoma also shows membrane-like material deposition but lacks dentinoid or dentino-osteoid differentiation and an ameloblastoma-like epithelial nest [19]. These findings are unlike those of the present case.

To date, only one case of extra-oral DGCT has been reported, and it occurred in the ethmoid sinus of an 8-year-old boy [3]. It exhibited odontogenic epithelium proliferation with ghost cells but lacked anatomic association with the oral and alveolar mucosa, as in the present case. The etiology of the DGCT remains unclear. However, the development of DGCT was thought to be de novo or from a preceding COC [3–5, 22], and the peripheral DGCT can originate from the oral epithelium following trauma or exposure to an irritating agent [14, 22]. The lesion in the present case did not have a history of odontogenic tumour, trauma, or surgery that may have caused tumour dissemination or metastasis. Furthermore, the lesion had no connection with the oral mucosa according to the results of the radiological and intraoperative examinations, and the cytological, histological, and genetic features were consistent with DGCT. Therefore, the ectopic odontogenic epithelium may have been associated with tumour development. Based on this clinical, pathological, and genetic evidence, a final diagnosis of extraosseous ectopic DGCT on the floor of the mouth was confirmed.

The recurrence rates of central and peripheral DGCT are 73% and 0%, respectively [1]. A simple excision is most often performed for both central and peripheral DGCT; however, resection is often performed in central DGCT [15]. As ectopic DGCT is extremely rare, the tumour aggressiveness and optimal treatment are unknown. Liu et al. [3] reported no recurrence of ectopic DGCT arising from the ethmoid sinus after endoscopic sinus surgery, during a 2-year follow-up. Similar to our findings, they observed that the Ki-67-labeling index was not high, and there was no invasion of the adjacent tissue or vascular and perineural structures, which suggests a low malignancy potential for ectopic DGCT. Therefore, a simple excision of the tumour is justified; however, further studies are required to clarify the nature of the tumour.

To the best of our knowledge, this is the first report of ectopic DGCT that has been diagnosed based on meticulous and comprehensive clinicopathological evidence. The nature of ectopic DGCT remains unknown owing to its rarity. Therefore, further studies are required to recognise ectopic DGCT as a new entity and elucidate its characteristic odontogenic features, which will help facilitate an accurate diagnosis and avoid overtreatment.

## Data Availability

Not applicable.

## Abbreviations

COC: Calcifying odontogenic cyst
DGCT: Dentinogenic ghost cell tumour
GCOC: Ghost cell odontogenic carcinoma
HE: Hematoxylin and eosin
MRI: Magnetic resonance imaging
